# Positive association between serum lactate dehydrogenase levels and blood pressure: evidence from NHANES 2015–2016

**DOI:** 10.3389/fcvm.2025.1554702

**Published:** 2025-02-28

**Authors:** Tao Hu, Linfeng Li, Qiqiang Cao, Weiling Tu, XianTao Huang, Tan Yuan

**Affiliations:** Department of Cardiology, Jiangxi Provincial People’s Hospital, The First Affiliated Hospital of Nanchang Medical College, Nanchang, Jiangxi, China

**Keywords:** serum lactate dehydrogenase, nutritional survey, blood pressure, multiple regression analysis, smooth curve fitting

## Abstract

**Background:**

Serum lactate dehydrogenase (sLDH) is an enzyme implicated in tissue injury and inflammatory responses. Despite its established role in these pathophysiological processes, the association between sLDH and blood pressure remains underexplored. The present findings suggest that sLDH could emerge as a valuable biomarker for blood pressure regulation and may hold significant promise in the management of hypertension.

**Methods:**

Our investigation utilized data from the National Health and Nutrition Examination Survey (NHANES) 2015–2016, comprising 3,469 participants after excluding those under the age of 20, individuals on antihypertensive therapies, and cases with incomplete data. sLDH levels were categorized into tertiles, while blood pressure measurements were conducted under standardized protocols. To elucidate the relationship between sLDH levels and blood pressure, multivariate regression analyses and smooth curve fitting techniques were employed, adjusting for 17 covariates, including age, sex, and body mass index.

**Results:**

sLDH corresponds with both systolic blood pressure (SBP) and diastolic blood pressure (DBP). The adjusted smooth curve fitting diagram demonstrates a linear positive connection between sLDH and SBP, with an increment of 0.053 mmHg (95% CI: 0.032, 0.074; *p* < 0.001) in SBP for every 1 U/L increment in LDH concentrations. The connection between sLDH and DBP is non-linear. sLDH concentrations below 123 U/L have a linear positive connection with DBP, increasing 0.079 mmHg (95% CI: 0.042, 0.115, *p* < 0.001). When sLDH concentrations exceed 123 U/L, there is not a substantial connection with DBP (*P* = 0.574).

**Conclusion:**

Our study demonstrates a linear positive correlation between sLDH and SBP. A non-linear association was observed between sLDH and DBP, with a positive relationship for sLDH levels below 123 U/L. These findings underscore the potential of sLDH as a biomarker for blood pressure regulation.

## 1 Introduction

Serum sactate dehydrogenase (sLDH) is a crucial enzyme found throughout numerous tissues in humans, including the heart, kidneys, lungs, liver, and other organs (1, 2). Elevated sLDH is a sensitive marker of enhanced cell membrane permeability and damage. An increase in sLDH levels may trigger a variety of illnesses, including hypertension, aortic dissection (3), diabetic nephropathy (4) and inflammatory disorders (5). A study revealed that sLDH concentrations were markedly elevated in pregnant women suffering from hypertensive disorders compared to their normotensive counterparts. Specifically, the mean sLDH concentrations were found to be 932 ± 448 U/L in women with severe preeclampsia, 1,515.86 ± 754 U/L in those with eclampsia, and 378.6 ± 124 U/L in normotensive women (6). Furthermore, another investigation demonstrated that LDH concentrations exceeding 400 U/L (1.6 times the normal reference value) were more prevalent among women with severe preeclampsia. Elevated LDH levels of ≥400 U/L were strongly correlated with unfavorable maternal outcomes (41.7% vs. 15.3%) and negative neonatal consequences, including preterm birth (59.4% vs. 22.5%) (7) An investigation carried out on women with preeclampsia at Liaquat University of Medical and Health Sciences demonstrated substantial differences in the average concentrations of sLDH between women with moderate and severe preeclampsia, with sLDH serving as a predictive marker for the severity of preeclampsia (8). An analysis of 1,507 arterial hypertension patients in China demonstrated that sLDH concentrations have been connected with flow-mediated dilation in hypertensive individuals (9). However, there is limited knowledge concerning the exact connection between sLDH and blood pressure. Emerging evidence suggests that elevated sLDH may contribute to hypertension via oxidative stress-induced endothelial dysfunction (10). Increased sLDH activity promotes lactate accumulation in the vascular microenvironment, activating NADPH oxidase in endothelial cells. This leads to excessive reactive oxygen species (ROS) production, which oxidizes tetrahydrobiopterin (BH4), uncoupling endothelial nitric oxide synthase (eNOS) (11–13). As a result, nitric oxide (NO) bioavailability decreases, and superoxide anion (O^2−^) levels rise, impairing vasodilation and increasing arterial stiffness. Chronic ROS overproduction may also induce mitochondrial dysfunction, creating a feedback loop that exacerbates oxidative damage (14, 15). This mechanism may explain the link between elevated sLDH levels and endothelial dysfunction markers, such as reduced flow-mediated dilation (FMD) and increased pulse wave velocity (PWV) in hypertensive patients. However, large-scale translational studies confirming this pathway in human populations are still limited.

Building upon these findings, the present study endeavors to elucidate the relationship between sLDH levels and blood pressure, leveraging data from the National Health and Nutrition Examination Survey (NHANES) 2015–2016 cycle. NHANES is an extensive, nationally representative dataset that furnishes invaluable health information from a diverse and heterogeneous cohort across the United States. It serves as a critical resource for probing the intricate relationship between sLDH and blood pressure, providing a wealth of demographic, clinical, and behavioral data that can be harnessed to investigate this association within a large-scale population.

This study aims to explore the manner in which variations in sLDH concentrations correlate with both systolic and diastolic blood pressure, with a particular focus on identifying potential threshold effects across various demographic and clinical subgroups. In doing so, we seek to illuminate the underlying role of sLDH in the regulation of blood pressure and assess its viability as a biomarker for predicting hypertension risk.

## 2 Materials and methods

### 2.1 Data source and sample population

**Figure 1:**
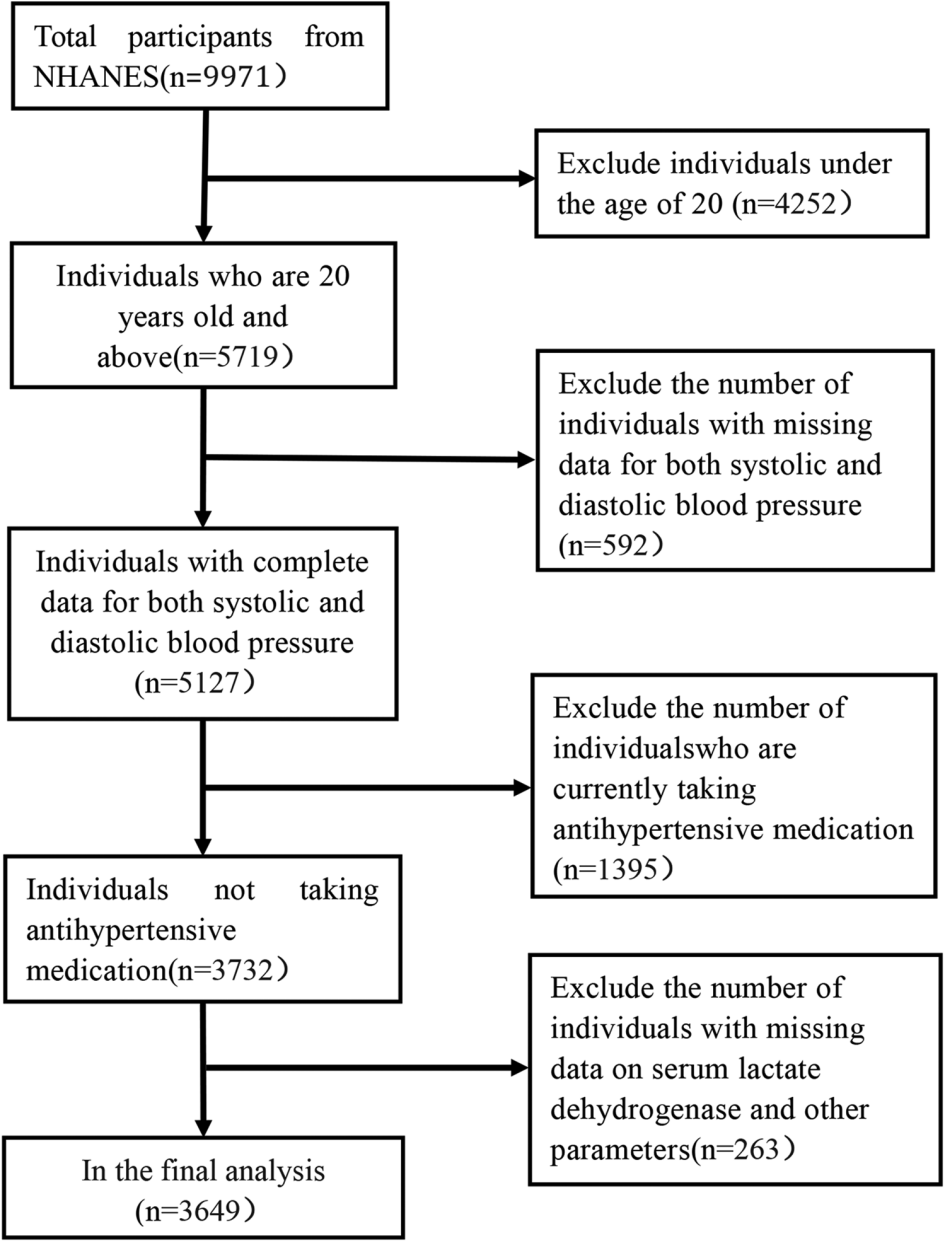
Flowchart of participant selection from NHANES 2015–2016.

The research individuals were drawn from the NHANES database (https://www.cdc.gov/nchs/nhanes/). NHANES endeavors to acquire knowledge regarding nutrition data of individuals in order to gauge their state of wellness. The study we are conducting is based on demographic data, examination test data, and questionnaire surveys collected from 2015 to 2016, representing one cycle of the NHANES database. All variables were collected simultaneously as part of the NHANES 2015–2016 cycle to ensure consistency and minimize biases associated with temporal variations. Our investigation had a total of 9,971 individuals; to capture adult data, we removed those under the age of 20. We also eliminated people who were taking antihypertensive drugs to ensure that the accuracy of the measured blood pressure data and sLDH data hadn't been affected by blood pressure-lowering medications. However, the remaining population comprised a large number of hypertension individuals who were not using antihypertensive drugs. Furthermore, people with incomplete data on body mass index (BMI), serum chloride concentrations, urea nitrogen, uric acid, and other variables were wiped out. We also omitted patients whose pre-data collection activities could have influenced the study's results. Finally, the investigation we conducted comprised 3,469 people (Figure 1).

### 2.2 Variables

The exposure variable in the current research is sLDH (U/L), based on their sLDH levels, all individuals were split into three groups: the lowest tertile, the middle tertile, and the highest tertile. Serum LDH was characterized using the enzymatic rate method using lactate as a substrate and the DxC800 reagent. The outcome variable is blood pressure (mmHg), which comprises both SBP and DBP. Blood pressure (BP) was measured according to standardized protocols provided by the NHANES program. Specifically, BP measurements were taken on the participant's right arm after the participant had been seated quietly for 5 min. Each participant's BP was measured three times, with approximately 1 min between measurements. The measurements were performed using a calibrated mercury sphygmomanometer, following the standardized procedure outlined in the NHANES 2015–2016 protocols. The average of the last two measurements was used for the analysis. To get more reliable research results, 17 covariates were incorporated, including sex, age, albumin, total calcium, blood urea nitrogen, race, bicarbonate, chloride, sodium ions, smoking status, triglycerides, uric acid, marital status, creatinine, aspartate aminotransferase (AST), education level, and alanine aminotransferase (ALT).

### 2.3 Statistical data analysis

The type of analysis used in this study is multivariate linear regression analysis, which allows us to assess the relationships between sLDH levels and blood pressure outcomes. To assess distinctions among descriptive analysis, weighted *t*-tests for students (for continuous variables) or weighted chi-square tests (for categorical variables) were utilized for statistical analysis, with univariate analysis to affect SBP and DBP variables and a weighted multivariate linear regression model to evaluate the connection between sLDH concentration and SBP and DBP. The regression analysis generated three statistical models. Model I is not adjusted for variables. Model II is modified for age, BMI, race/Hispanic origin, and sex. Model III is adjusted for all relevant variables, such as sex, education level, smoking status, albumin, marital status, total calcium, age, blood urea nitrogen, bicarbonate, chloride, triglycerides, uric acid, race/Hispanic origin, creatinine, AST, and ALT. Smooth curve fitting and generalized additive models were utilized to examine the nonlinear connection among sLDH concentrations, SBP, and DBP. The *β* value represents the relative changes in blood pressure values between different sLDH groups. Specifically, the *β* value refers to the change in blood pressure for every 1 U/L increase in sLDH concentration. Each analysis was executed using the statistical software packages R and Empower Statistics. *P*-values <0.05 demonstrate statistical significance.

## 3 Results

### 3.1 Baseline characteristics across sLDH tertiles

The investigation comprised 3,469 individuals, with sLDH levels categorized into three tertiles: the lowest tertile (*n* = 1,122) (≥51–114 U/L), the middle tertile (*n* = 1,145) (≥114–133 U/L), and the highest tertile (*n* = 1,202) (≥133 to ≤309 U/L). The baseline characteristics of variables, including sex, marital status, body mass index (BMI), age, SBP, DBP, race/Hispanic origin, blood urea nitrogen, bicarbonate, chloride, triglycerides, uric acid, creatinine, aspartate aminotransferase (AST), and alanine aminotransferase (ALT), varied across the sLDH tertiles (*P* < 0.05). Specifically, sLDH levels were observed to increase with age, BMI, and other relevant variables. However, factors like education level, smoking status, albumin, total calcium, and sodium ions did not show significant associations with sLDH levels (*P* > 0.05). The results suggest that higher sLDH concentrations are associated with increased age and BMI (Table 1 and Supplementary Table S1).

**Table 1:** Baseline characteristics of participants (*N* = 3,469).

sLDH (U/L) tertile	Low group (*N* = 1,112)	Medium group (*N* = 1,155)	High group (*N* = 1,202)	*P*-value
Age (years)	40.3 ± 15.3	44.0 ± 16.2	48.3 ± 16.6	<0.001
BMI (kg/m^2^)	27.5 ± 6.1, 45 ± 4.154	28.6 ± 6.8	30.2 ± 7.1	<0.001
SBP (mmHg)	117 ± 14.5	121.5 ± 15.4	126 ± 17.4	<0.001
DBP (mmHg)	68.2 ± 10.7	70.7 ± 10.7	71.7 ± 12.2	<0.001
Blood urea nitrogen (mmol/L)	5.40 ± 2.13	5.28 ± 2.11	5.65 ± 2.42	<0.001
Serum albumin (g/L)	43.3 ± 3.39	43.5 ± 3.58	43.3 ± 3.38	0.371
Bicarbonate (mmol/L)	24.2 ± 2.14	24.3 ± 2.06	24.5 ± 2.12	0.009
Total calcium (mmol/L)	2.33 ± 0.08	2.34 ± 0.09	2.33 ± 0.09	0.300
Sodium (mmol/L)	138 ± 1.97	138 ± 01	138 ± 2.09	0.147
Chloride (mmol/L)	103 ± 2.58	103 ± 2.61	103 ± 2.68	<0.001
sLDH (U/L)	101 ± 9.46	122 ± 5.40	153 ± 20.89	<0.001
Triglycerides, refrig serum (mmol/L)	1.54 ± 1.15	1.73 ± 1.26	1.89 ± 1.54	<0.001
Uric acid (umol/L)	299 ± 77.31	311 ± 78.85	327 ± 81.68	<0.001
Creatinine (umol/L)	70.3 ± 21.74	72.1 ± 18.14	76.7 ± 27.06	<0.001
Aspartate aminotransferase AST (U/L)	21.6 ± 6.60	24.1 ± 7.85	29.9 ± 16.75	<0.001
Alanine aminotransferase ALT (U/L)	21.0 ± 11.54	24.2 ± 13.17	30.3 ± 21.79	<0.001
Gender				
Male	494 (44.42%)	561 (48.57%)	631 (52.50%)	<0.001
Female	618 (55.58%)	594 (51.43%)	571 (47.50%)	

Continuous variables are expressed as mean ± standard deviation; categorical variables are expressed as *n* (%). sLDH, serum lactate dehydrogenase.

### 3.2 Univariate and stratified analysis of sLDH and blood pressure

Table 2 demonstrates the outcomes of the single-variable analysis. The concentration of sLDH corresponds positively with SBP and DBP. In the research of SBP and DBP, utilizing the low tertile group of sLDH as the reference group, the beta values for both the middle tertile group of sLDH and the high tertile group of sLDH are more than 0, and the *P*-values are all less than 0.001 (Table 2 and Supplementary Table S2).The analysis revealed that the middle sLDH tertile had higher systolic blood pressure (SBP) by 3.95 mmHg (*β* = 3.95, 95% CI: 2.64, 5.25; *P* < 0.001) and diastolic blood pressure (DBP) by 2.53 mmHg (*β* = 2.53, 95% CI: 1.60, 3.45; *P* < 0.001) compared to the lowest sLDH tertile. The highest sLDH tertile exhibited even higher SBP, with an increase of 8.407 mmHg (*β* = 8.41, 95% CI: 7.10, 9.69; *P* < 0.001), and DBP, with an increase of 3.47 mmHg (*β* = 3.47, 95% CI: 2.56, 4.39; *P* < 0.001), compared to the lowest sLDH tertile (Table 2 and Supplementary Table S2). SBP and DBP are additionally connected to sex, age, education level, smoking status, albumin, race/Hispanic origin, total calcium, chloride, triglycerides, uric acid, creatinine, marital status, and AST. Blood urea nitrogen and bicarbonate concentrations are connected with SBP but not significantly to DBP. Blood concentrations of sodium are not significantly connected to either SBP or DBP. To delve deeper, stratified analysis was implemented (as indicated in Supplementary Table S3). In the examination of baseline SBP, sLDH has a positive connection with SBP in practically all strata except for the widowed population group. In the analysis of DBP, sLDH is positively correlated with DBP in most strata; in the stratification by race/Hispanic origin, the beta value is the highest in the middle tertile group of sLDH among non-Hispanic and non-Hispanic white populations; in the smoking stratification, the beta value is the highest in the middle tertile of sLDH among smokers.

**Table 2:** Univariate analysis of systolic and diastolic blood pressure.

Variable	Statistics	SBP (mmHg)		DBP (mmHg)	
		β (95% CI)	*p*-value	β (95% CI)	*p*-value
sLDH (mmol/L)	126 ± 25.4	0.14 (0.11, 0.16)	<0.001	0.06 (0.04, 0.07)	<0.001
sLDH (mmol/L)					
Low	1,112 (32.06%)	0		0	
Medium	1,155 (33.29%)	3.95 (2.64, 5.25)	<0.001	2.53 (1.60, 3.45)	<0.001
High	1,202 (34.65%)	8.41 (7.10, 9.69)	<0.001	3.47 (2.56, 4.39)	<0.001

Continuous variables are expressed as mean ± SD; categorical variables are expressed as *n* (%). The first group serves as the reference for each univariate analysis group (*β* = 0); (a) includes multiracial; (b) includes 12th grade, no diploma; (c) GED or equivalent. sLDH, serum lactate dehydrogenase; SBP, systolic blood pressure; DBP, diastolic blood pressure; β, beta value, CI, confidence interval.

### 3.3 Multivariate regression analysis of sLDH and blood pressure

The findings of the multiple regression analysis are outlined in Table 3. In unadjusted Model I, sLDH concentrations are positively linked with SBP (*β* = 0.136, 95% CI: 0.115, 0.156, *P* < 0.00001) and DBP (*β* = 0.057, 95% CI: 0.042, 0.071, *P* < 0.001), or each 10 U/L increase in LDH, there is an increase of 1.36 mmHg in SBP and 0.57 mmHg in DBPModels II (Beta = 0.078, 95% CI: 0.059, 0.098, *P* < 0.001) (*β* = 0.048, 95% CI: 0.033, 0.063, *P* < 0.00001) and III (*β* = 0.053, 95% CI: 0.032, 0.074, *P* < 0.001) (*β* = 0.031, 95% CI: 0.015, 0.047, *P* = 0.001) maintain the positive connection after controlling for confounding factors, for each 10 U/L increase in sLDH, there is an increase of 0.78 mmHg in SBP and 0.48 mmHg in DBP. Furthermore, in the analysis of sLDH with SBP, utilizing the low concentration category as a basis for comparison, the beta values of the high concentration group are substantially higher than those of the medium concentration group in all models. The beta values rise with the rise of sLDH content. In the analysis of baseline DSP, with the low concentration group as the reference, the beta values of the high concentration group are substantially higher than those of the medium concentration group in Models I and II, but in Model III, the beta value of the high concentration group is lower than that of the medium concentration group.

**Table 3:** The relationship between Serum lactate dehydrogenase and blood pressure (multivariate regression equation analysis).

Results	Model I		Model II		Model III	
	β (95% CI)	*p*-value	β (95% CI)	*p*-value	β (95% CI)	*p*-value
Y = SBP (mmHg)						
sLDH (U/L)	0.136 (0.115, 0.156)	<0.001	0.078 (0.059, 0.098)	<0.001	0.053 (0.032, 0.074)	<0.001
sLDH (U/L) tertile						
Low	0		0		0	
Middle	3.95 (2.64, 5.25)	<0.001	2.31 (1.14, 3.50)	<0.001	1.57 (0.41, 2.74)	0.008
High	8.39 (7.104, 9.689)	<0.001	4.60 (0.77, 1.22)	<0.001	2.88 (1.63, 4.13)	<0.001
Y = DBP (mmHg)						
sLDH (U/L)	0.057 (0.042, 0.071)	<0.001	0.048 (0.033, 0.063)	<0.001	0.031 (0.015, 0.047)	<0.001
sLDH (U/L) tertile						
Low	0		0		0	
Middle	2.52 (1.60, 3.45)	<0.001	2.35 (1.43, 3.27)	<0.001	1.75 (0.85, 2.66)	0.014
High	3.47 (2.58, 4.39)	<0.001	2.94 (2.01, 3.87)	<0.001	1.71 (0.74, 2.68)	<0.001

SBP, systolic blood pressure; DBP, diastolic blood pressure; β, beta value; CI, confidence interval. Weighting basis: Complete sample with mobile examination center examination weights. Outcome variables: Baseline SBP; Baseline DBP. Exposure variable: Lactate Dehydrogenase (U/L). Model I is not adjusted for variables. Model II is modified for age, race/Hispanic origin, and sex. Model III is adjusted for all relevant variables, such as sex, education level, smoking status, albumin, marital status, total calcium, age, blood urea nitrogen, bicarbonate, chloride, triglycerides, uric acid, race/Hispanic origin, creatinine, aspartate aminotransferase (AST), and alanine aminotransferase (ALT). sLDH, serum lactate dehydrogenase.

### 3.4 Smooth curve fitting and threshold analysis of sLDH and blood pressure

**Figure 2:**
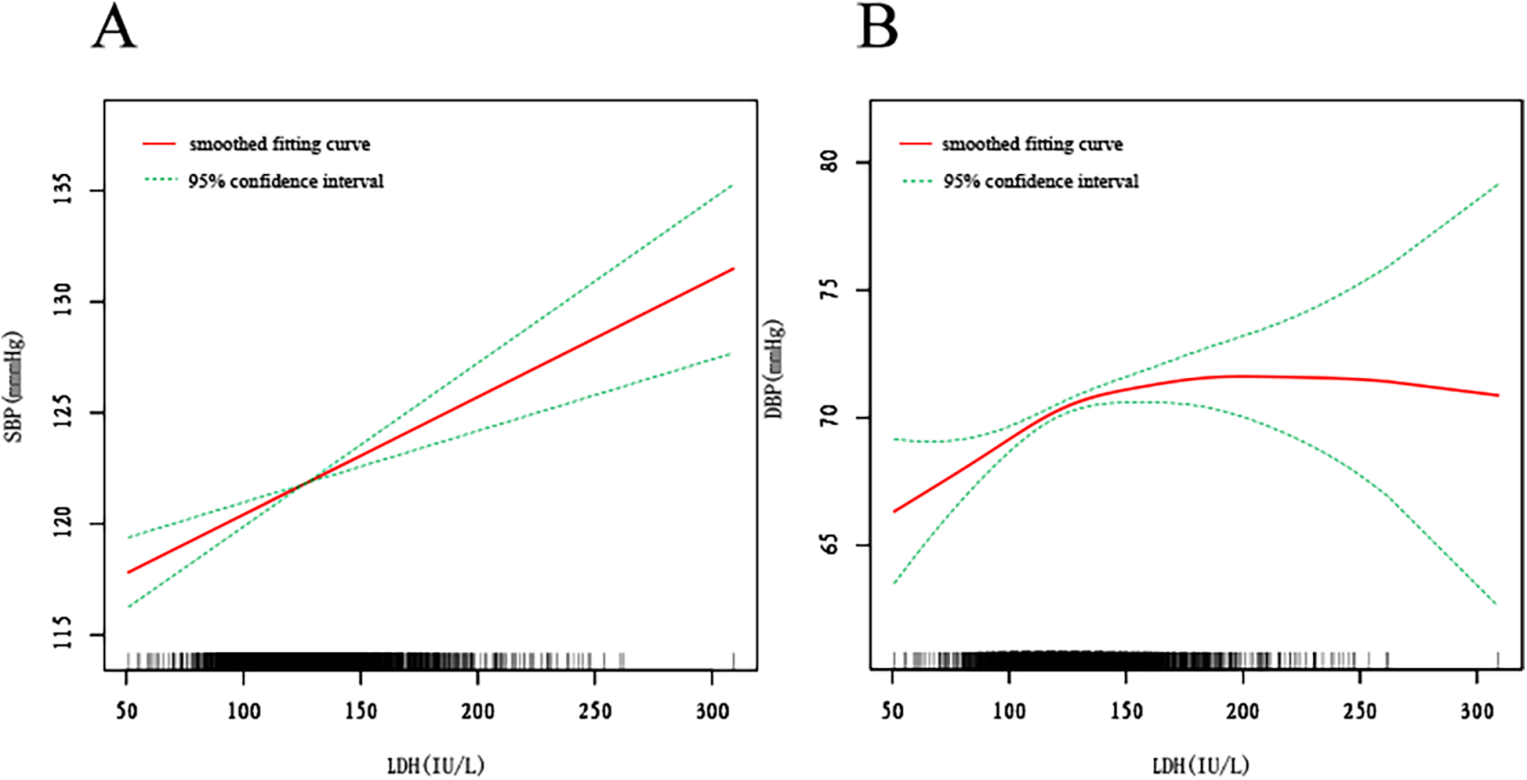
The smooth fitting curve between serum lactate dehydrogenase (sLDH) and systolic blood pressure (SBP), diastolic blood pressure (DBP). The smooth curve fitting is represented by the red line, and the fitted 95% confidence interval is represented by the green line. Weighting basis: Complete sample with mobile examination center examination weights. Adjusted for age (smoothed), sex, race/Hispanic origin, marital status, Education level-Adults 20+, smoking (yes, no), body mass index (BMI) (smoothed), albumin (smoothed), blood urea nitrogen (smoothed), bicarbonate (smoothed), total calcium (smoothed), chloride (smoothed), triglycerides (smoothed), uric acid (smoothed), creatinine (smoothed), aspartate aminotransferase (smoothed), alanine aminotransferase (smoothed). **(A)** Smooth curve fitting plot of baseline LDH and SBP. **(B)** Smooth curve fitting plot of baseline LDH and DBP.

We additionally utilized smooth curve fitting (Figure 2) and threshold and saturation effect analysis (Supplementary Table S4) to ascertain the connection among DBP, SBP, and sLDH. Supplementary Table S4 summarizes the covariates utilized for adjustment. Figure 2A demonstrates a linear and positive connection between LDH and SBP, with a rise of 1 U/L in sLDH concentration resulting to an increase of 0.053 mmHg in SBP (*β* = 0.053, 95% CI: 0.032, 0.074, *P* < 0.001) (Figure 2A, Supplementary Table S4). Figure 2B demonstrates the connection between sLDH and DBP. When LDH is less than 123 U/L, it is linearly and positively connected with DBP. An increase of 1 U/L in LDH concentration leads to a rise of 0.079 mmHg in SBP (*β* = 0.079, 95% CI: 0.042, 0.115, *P* < 0.001). When sLDH concentrations exceed 123 U/L, there is not a significant connection between sLDH and DBP (*P* = 0.574) (Figure 2B, Supplementary Table S4). Smooth fitting curves were presented for six covariates: sex, education level, age, marital status, race/Hispanic origin, and smoking status (Supplementary Figures S3, S4). In the smooth curve analysis describing the connection between sLDH and SBP (Supplementary Figure S1), most stratified populations demonstrate an increase in SBP with a rise in sLDH, while the fitting curves in other races and married populations exhibit considerable fluctuation. In males, sLDH increases with SBP initially, but as sLDH reaches 155, it starts to decrease with increasing SBP. Additionally, in the non-smoking population, the rate of increase in sLDH with SBP is higher than in the smoking population. In the smooth curve analysis of sLDH and DBP (Supplementary Figure S2), many stratified populations show an increase in DBP with an increase in sLDH, while the fitting curves in male populations and widowed populations exhibit considerable fluctuation. In individuals aged ≥60 years, when sLDH exceeds 175 U/L, DBP begins to decrease with increasing sLDH. A similar phenomenon was observed in the smoking population, with the threshold at 130 U/L. Furthermore, in Mexican Americans, there is a proportional relationship between sLDH and DBP.

## 4 Discussion

Hypertension is a significant contributor of cardiovascular diseases, which threaten worldwide health (16). Long-term hypertension may trigger both macrovascular disorders (aortic dissection, heart failure, and stroke) and microvascular disorders (nephropathy and retinopathy) (17–20). Elevated blood pressure is the largest preventable cause of cardiovascular disease mortality and disease burden worldwide, as well as in the majority of areas (16, 21).

This study meticulously examines the relationship between sLDH levels and blood pressure, uncovering a substantial association between elevated sLDH and heightened SBP and DBP. Our findings corroborate prior research linking higher sLDH concentrations to increased cardiovascular risk, particularly within hypertensive cohorts. In our analysis, we observed a distinct and linear positive correlation between sLDH levels and both SBP and DBP. Specifically, individuals in the highest tertile of sLDH displayed an increase of 3.47 mmHg in DBP and 8.41 mmHg in SBP when compared to those in the lowest tertile. These results are in alignment with previous studies, such as those by Burwick et al. (6), which revealed elevated sLDH levels in preeclamptic patients, correlating with adverse maternal and fetal outcomes, including elevated blood pressure. In their work, the authors posited that elevated sLDH could serve as a pivotal biomarker for hypertensive disorders during pregnancy, reflecting the extent of vascular damage and dysfunction. Similarly, our study reinforces this observation, demonstrating a consistent positive relationship between sLDH and blood pressure, with significance maintained across various subgroups, including those stratified by age, smoking status, and race. Moreover, our multivariate analysis further substantiates the association between sLDH and blood pressure. After adjusting for confounding variables such as age, BMI, and smoking status, the relationship persisted, with each 10 U/L increase in sLDH correlating with a 0.78 mmHg increase in SBP and a 0.48 mmHg rise in DBP. These results are in accordance with the findings of Reddy et al., which demonstrated that elevated sLDH levels are significantly associated with both increased blood pressure and the severity of preeclampsia. The robustness of this association across multiple models lends further credence to the potential of sLDH as a valuable biomarker in the management of hypertension. Intriguingly, our smooth curve fitting analysis unveiled that sLDH maintains a linear relationship with DBP when concentrations are below 123 U/L, yet this association weakens and becomes statistically insignificant when sLDH concentrations surpass this threshold. This observation suggests the presence of a saturation effect, where further increases in sLDH do not yield additional changes in DBP beyond a specific point. This nonlinear relationship with DBP remains an underexplored avenue in existing literature and warrants further research to elucidate the underlying mechanisms.

Elevated sLDH is a sensitive marker of enhanced cell membrane permeability and damage (22). An increase in sLDH levels may trigger a variety of illnesses, including malignant tumors (23, 24), aortic dissection (3), diabetic nephropathy (4), inflammatory disorders (5), and chronic obstructive pulmonary disease (25). Some investigations have discovered a connection between sLDH and disease-related renal involvement. Cai X et al. demonstrated that elevated sLDH levels are an early indicator of a higher likelihood of renal dysfunction (26). Zager RA et al. demonstrated that sLDH is released from cells during the damage-related process, which renders it a possible sign of kidney damage (27). Uslu S et al. discovered that urine sLDH activity is highly positively connected with serum creatinine and that urinary sLDH activity begins to increase as creatinine clearance declines (28). The kidney plays a crucial role for blood pressure regulation, and chronic kidney damage can activate the renin-angiotensin-aldosterone system (RAAS), resulting in increased resistance in the kidney's afferent arterioles, reduced renal blood flow and glomerular filtration rate, and, ultimately, elevated blood pressure (29). Emerging evidence suggests that sLDH elevation may contribute to hypertension through oxidative stress-mediated endothelial dysfunction (10). Elevated sLDH activity promotes lactate accumulation in the vascular microenvironment, which acts as a potent activator of NADPH oxidase in endothelial cells. This activation triggers excessive ROS production, subsequently oxidizing the essential cofactor tetrahydrobiopterin (BH4) and uncoupling eNOS (11). Depleted NO bioavailability combined with increased superoxide anion (O^2−^) generation leads to impaired endothelium-dependent vasodilation and increased arterial stiffness. Additionally, chronic ROS overproduction may induce mitochondrial dysfunction in vascular endothelial cells, creating a vicious cycle that perpetuates oxidative damage and exacerbates hemodynamic dysregulation (14). This pathophysiological cascade potentially explains the observed association between sLDH levels and clinical markers of endothelial dysfunction, including reduced FMD and elevated PWV in hypertensive cohorts. However, translational studies verifying this mechanistic pathway in large human populations remain limited.

The primary goal of this study is to evaluate the connection between sLDH and blood pressure in a nationally representative sample of American adults (*n* = 3,469). Our research has numerous advantages: (1) a large sample size and a diverse study population; (2) rigorous statistical approaches that reduce the impact of confounding variables. The findings demonstrate that sLDH has a positive connection with both SBP and DBP and that a rise in sLDH frequently indicates an increase in blood pressure. When clinicians notice variations in sLDH, they should often monitor and manage blood pressure fluctuations to avoid serious implications and problems.

This research has several limitations. First, the study sample was limited to American individuals, and thus the results may not be applicable to other populations or regions, particularly those with a higher prevalence of certain races known to have higher sLDH levels and more refractory hypertension. Furthermore, the study's cross-sectional design only allows for the assessment of sLDH concentrations at a single time point, and as such, it is not possible to establish a causal relationship or determine the temporal connection between sLDH levels and blood pressure. Follow-up studies over longer periods of time are recommended to address this limitation. Additionally, the biochemical mechanisms by which sLDH affects blood pressure remain unclear and require further investigation. Despite controlling for multiple confounders using statistical methods, the presence of other unmeasured confounding factors cannot be completely ruled out. Finally, due to the lack of data on sLDH isozymes in the NHANES database, we were unable to explore how changes in sLDH isozyme levels may impact the relationship between sLDH and blood pressure. Further studies incorporating sLDH isozyme analysis may provide additional insights.

## 5 Conclusion

Our study demonstrates a linear positive correlation between sLDH and SBP. A non-linear association was observed between sLDH and DBP, with a positive relationship for sLDH levels below 123 U/L. These findings underscore the potential of sLDH as a biomarker for blood pressure regulation.

## Data Availability

Publicly available datasets were analyzed in this study. This data can be found here: https://www.cdc.gov/nchs/nhanes/index.htm.

## Supplementary material

Supplementary Figure S1 The smooth fitting curve between serum lactate dehydrogenase (sLDH) and systolic blood pressure (SBP).Stratified by covariates (sex, age, race/Hispanic origin, marital status, Education level-Adults 20+, smoking status). Adjusted for age (smoothed), sex, race/Hispanic origin, marital status, Education level-Adults 20+, smoking (yes, no), body mass index (BMI) (smoothed), albumin (smoothed), blood urea nitrogen (smoothed), bicarbonate (smoothed), total calcium (smoothed), chloride (smoothed), triglycerides (smoothed), uric acid (smoothed), creatinine (smoothed), aspartate aminotransferase (smoothed), alanine aminotransferase (smoothed). **(A)** Stratified by sex. **(B)** Stratified by age. **(C)** Stratified by race/Hispanic origin. **(D)** Stratified by Education level-Adults 20+. **(E)** Stratified by marital status. **(F)** Stratified by smoking status.

Supplementary Figure S2 The smooth fitting curve between serum lactate dehydrogenase (sLDH) and diastolic Blood Pressure (DBP). Stratified by covariates (sex, age, race/Hispanic origin, marital status, Education level-Adults 20+, smoking status). Adjusted for age (smoothed), sex, race/Hispanic origin, marital status, Education level-Adults 20+, smoking (yes, no), body mass index (BMI) (smoothed), albumin (smoothed), blood urea nitrogen (smoothed), bicarbonate (smoothed), total calcium (smoothed), chloride (smoothed), triglycerides (smoothed), uric acid (smoothed), creatinine (smoothed), aspartate aminotransferase (smoothed), alanine aminotransferase (smoothed). **(A)** Stratified by sex. **(B)** Stratified by age. **(C)** Stratified by race/Hispanic origin. **(D)** Stratified by Education level-Adults 20+. **(E)** Stratified by marital status. **(F)** Stratified by smoking status.
