# Supplementary figures and images for: Positive association between serum lactate dehydrogenase levels and blood pressure: evidence from NHANES 2015–2016
Source: Front Cardiovasc Med. 2025 Feb 28;12:1554702. doi: 10.3389/fcvm.2025.1554702 (PMC11906999; doi:10.3389/fcvm.2025.1554702)

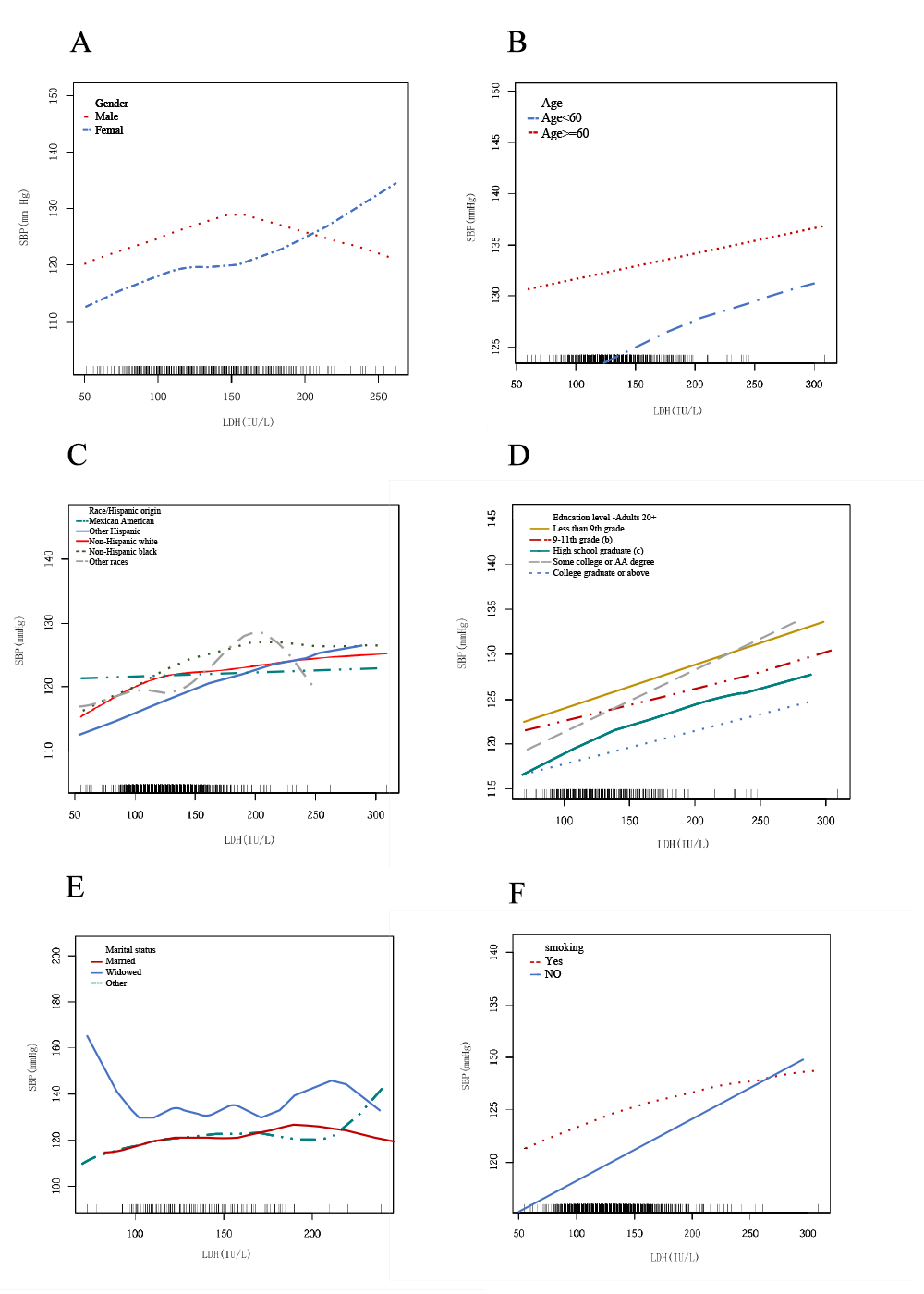

Supplement: Supplementary Figure S1 — The smooth fitting curve between serum lactate dehydrogenase (sLDH) and systolic blood pressure (SBP).Stratified by covariates (sex, age, race/Hispanic origin, marital status, Education level-Adults 20+, smoking status). Adjusted for age (smoothed), sex, race/Hispanic origin, marital status, Education level-Adults 20+, smoking (yes, no), body mass index (BMI) (smoothed), albumin (smoothed), blood urea nitrogen (smoothed), bicarbonate (smoothed), total calcium (smoothed), chloride (smoothed), triglycerides (smoothed), uric acid (smoothed), creatinine (smoothed), aspartate aminotransferase (smoothed), alanine aminotransferase (smoothed). (A) Stratified by sex. (B) Stratified by age. (C) Stratified by race/Hispanic origin. (D) Stratified by Education level-Adults 20+. (E) Stratified by marital status. (F) Stratified by smoking status. [file Image1.tif]

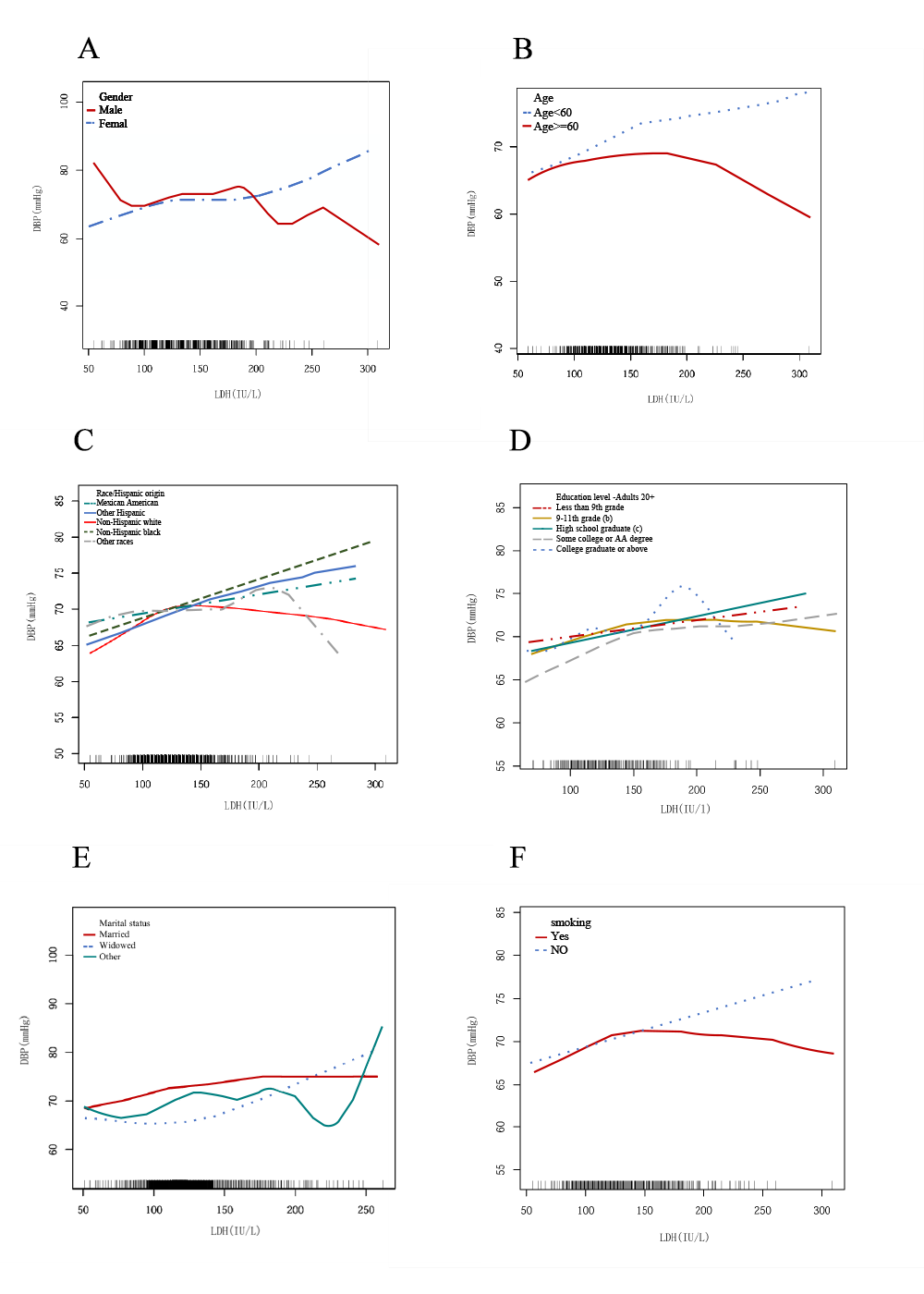

Supplement: Supplementary Figure S2 — The smooth fitting curve between serum lactate dehydrogenase (sLDH) and diastolic Blood Pressure (DBP). Stratified by covariates (sex, age, race/Hispanic origin, marital status, Education level-Adults 20+, smoking status). Adjusted for age (smoothed), sex, race/Hispanic origin, marital status, Education level-Adults 20+, smoking (yes, no), body mass index (BMI) (smoothed), albumin (smoothed), blood urea nitrogen (smoothed), bicarbonate (smoothed), total calcium (smoothed), chloride (smoothed), triglycerides (smoothed), uric acid (smoothed), creatinine (smoothed), aspartate aminotransferase (smoothed), alanine aminotransferase (smoothed). (A) Stratified by sex. (B) Stratified by age. (C) Stratified by race/Hispanic origin. (D) Stratified by Education level-Adults 20+. (E) Stratified by marital status. (F) Stratified by smoking status. [file Image2.tif]
