# Supplementary material for: Positive association between serum lactate dehydrogenase levels and blood pressure: evidence from NHANES 2015–2016
Source: Front Cardiovasc Med. 2025 Feb 28;12:1554702. doi: 10.3389/fcvm.2025.1554702 (PMC11906999; doi:10.3389/fcvm.2025.1554702)
Supplement: Supplementary file 3 [file Table1.docx]

**Table S1. Continuation of Baseline Characteristics of Participants (N=3469)**

| **Serum Lactate Dehydrogenase(IU/L) Tertile** | **Low Group**  **(N=1112)** | **Medium Group**  **(N=1155)** | **High Group (N=1202)** | **P-value** |
| --- | --- | --- | --- | --- |
| Race/Hispanic Origin |  |  |  | <0.001 |
| Mexican American | 234（21.04%） | 219（18.96%） | 203（16.89%） |  |
| Other Hispanic | 148（13.31%） | 152（13.16%） | 168（13.98%） |  |
| Non-Hispanic White | 352（31.65%） | 425（36.80%） | 381（31.70%） |  |
| Non-Hispanic Black | 176（15.83%） | 180（15.58%） | 259（21.55%） |  |
| Other Races | 202（18.17%） | 179（16.50%） | 191（15.89%） |  |
| Education level -Adults 20+ |  |  |  | 0.196 |
| Less than 9th grade | 101（9.08%） | 126（10.91%） | 143（11.90%） |  |
| 9-11th grade (b) | 122（10.97%） | 123（10.65%） | 140（11.65%） |  |
| High school graduate (c) | 229（20.59%） | 244（21.13%） | 268（22.30%） |  |
| Some college or AA degree | 333（29.95%） | 354（30.65%） | 356（29.62%） |  |
| College graduate or above | 327（29.41%） | 308（26.67%） | 295（24.54%） |  |
| Marital Status |  |  |  | 0.003 |
| Married | 546（49.10%） | 611（52.90%） | 626（52.08%） |  |
| Widowed | 34（3.06%） | 43（3.72%） | 67（5.57%） |  |
| Other | 532（47.84%） | 501（43.38%） | 509（42.35%） |  |
| Smoking |  |  |  | 0.125 |
| Yes | 417（37.50%） | 472（40.87%） | 497（41.35%） |  |
| No | 695（62.50%） | 683（59.13%） | 705（58.65%） |  |

Note: Continuous variables are expressed as mean ± standard deviation; categorical variables are expressed as n (%).
