# Supplementary material for: Positive association between serum lactate dehydrogenase levels and blood pressure: evidence from NHANES 2015–2016
Source: Front Cardiovasc Med. 2025 Feb 28;12:1554702. doi: 10.3389/fcvm.2025.1554702 (PMC11906999; doi:10.3389/fcvm.2025.1554702)
Supplement: Supplementary file 4 [file Table2.docx]

**Table S2. Continuaiton of univariate Analysis of Systolic and Diastolic Blood Pressure**

| **Variable** | **Statistics** | **SBP(mmHg)**  **β(95%CI) *p*-value** | **DBP(mmHg)**  **β(95%CI) *p*-value** |
| --- | --- | --- | --- |
| Albumin (g/L) | 43.38±3.45 | -0.25（-0.41,-0.10） 0.002 | 0.27（0.16,0.38） <0.001 |
| Albumin (g/L) Tertile |  |  |  |
| Low | 950（27.39%） | 0 | 0 |
| Medium | 1165（33.58%） | -1.28（-2.67,0.11） 0.070 | 1.09（0.12,2.06） 0.028 |
| High | 1354（39.03%） | -2.26（-3.60,-0.92）0.001 | 1.86（0.92,2.80） <0.001 |
| Blood Urea Nitrogen (mmol/L) | 4.88±1.57 | 1.51（1.17,1.85） <0.001 | 0.13（-0.11,0.37）0.275 |
| Blood Urea Nitrogen (mmol/L) Tertile |  |  |  |
| Low | 1135（32.72%） | 0 | 0 |
| Medium | 1021（29.43%） | 2.39（1.03，3.76） 0.006 | 0.77（-0.19，1.73） 0.115 |
| High | 1313（37.85%） | 4.28（3.00，5.56）<0.001 | 1.07（0.17，1.97） 0.020 |
| Bicarbonate (mmol/L) | 24.33±2.11 | 0.60（0.35,0.86） <0.001 | 0.04（-0.14,00.22）0.660 |
| Bicarbonate (mmol/L) Tertile |  |  |  |
| Low | 1152（33.21%） | 0 | 0 |
| Medium | 655（18.88%） | 1.57（0.02，3.13）0.047 | 0.16（-0.93，1.24）0.776 |
| High | 1662（47.91%） | 2.37（1.16，3.59）<0.001 | 0.19（-0.66，1.04）0.664 |
| Total calcium(mmol/L) | 2.33±0.08 | 8.17（1.78,14.55）0.0123 | 5.90（1.44，10.36）0.0096 |
| Totalcalcium(mmol/L)  Tertile |  |  |  |
| Low | 1085（31.26%） | 0 | 0 |
| Medium | 1227（35.37%） | -0.65（-1.97，0.68）0.3396 | -0.15（-1.07，0.78）0.7524 |
| High | 1157（33.35%） | 1.00（-0.34，2.34）0.1445 | 0.96（0.02，1.90）0.0450 |
| Sodium(mmol/L) | 138.70±2.02 | 0.25（-0.02，0.52）0.0669 | -0.14（-0.33，0.05）0.1376 |
| Sodium(mmol/L)  Tertile |  |  |  |
| Low | 878（25.31%） | 0 | 0 |
| Medium | 1416（40.82%） | 0.04（-1.33，1.40）0.9592 | -0.43（-1.39，0.52）0.3715 |
| High | 1175（33.87%） | 1.64（0.23，3.06）0.0230 | -0.86（-1.85，0.13）0.0877 |
| Chloride(mmol/L) | 103.59±2.63 | -0.49（-0.70，-0.29）<0.0001 | -0.32（-0.46，-0.18）<0.0001 |
| Chloride(mmol/L)Tertile |  |  |  |
| Low | 1113（32.08%） | 0 | 0 |
| Medium | 1046（30.15%） | -2.18（-3.55，-0.81）0.0018 | -0.64（-1.60，0.31）0.1877 |
| High | 1310（37.76%） | -2.24（-3.53，-0.94）0.0007 | -1.65（-2.55，-0.74）<0.0001 |
| Triglycerides,refrig serum(mmol/L) | 1.73±1.34 | 1.78（1.38，2.18）<0.0001 | 1.43（1.15，1.71）<0.0001 |
| Triglycerides,refrig serum(mmol/L) Tertile |  |  |  |
| Low | 1148（33.09%） | 0 | 0 |
| Medium | 1161（33.47%） | 3.44（2.13，4.74）<0.0001 | 1.40（0.49，2.31）0.0026 |
| High | 1160（33.44%） | 6.31（5.01，7.62）<0.0001 | 4.56（3.65，5.47）<0.0001 |
| Uric acid(umol/L) | 312.93±80.19 | 0.04（0.03，0.04）<0.0001 | 0.02（0.02，0.03）<0.0001 |
| Uric acid(umol/L)  Tertile |  |  |  |
| Low | 1139（32.83%） | 0 | 0 |
| Medium | 1171（33.76%） | 3.12（1.82，4.43）<0.0001 | 1.82（0.91，2.74）<0.0001 |
| High | 1159（33.41%） | 6.71（5.41，8.02）<0.0001 | 3.97（3.06，4.89）<0.0001 |
| Creatinine(umol/L) | 73.19±22.84 | 0.12（0.10，0.14）<0.0001 | 0.04（0.03，0.06）<0.0001 |
| Creatinine(umol/L)  Tertile |  |  |  |
| Low | 1133（32.66%） | 0 | 0 |
| Medium | 1154（33.27%） | 2.58（1.26，3.89）0.0001 | 1.59（0.66,2.51）0.0008 |
| High | 1182（34.07%） | 5.73（4.43，7.04）<0.0001 | 2.71（1.79,3.63）<0.0001 |
| Aspartate aminotransferase AST(U/L) | 25.33±12.00 | 0.14（0.09，0.18）<0.0001 | 0.08（0.05,0.12）<0.0001 |
| Aspartate aminotransferase AST(U/L) Tertile |  |  |  |
| Low | 1100（31.71%） | 0 | 0 |
| Medium | 1187（34.22%） | 3.15（1.83，4.47）<0.0001 | 1.90（0.97，2.82）<0.0001 |
| High | 1182（34.07%） | 5.65（4.33，6.97）<0.0001 | 3.51（2.58，4.43）<0.0001 |
| Alanine aminotransferase ALT(U/L) | 25.30±16.73 | 0.09（0.06,0.12）<0.0001 | 0.10（0.07，0.12）<0.0001 |
| Alanine aminotransferase ALT(U/L) Tertile |  |  |  |
| Low | 1011（29.14%） | 0 | 0 |
| Medium | 1212（34.94%） | 3.52（2.18，4.86）<0.0001 | 2.16（1.23，3.10）<0.0001 |
| High | 1246（35.92%） | 5.18（3.84，6.51）<0.0001 | 4.86（3.93，5.78）<0.0001 |
| BMI(kg/m^2^） | 1.149±0.165 | 0.966（0.675，1.383）0.851 | 0.966（0.675，1.383）0.851 |
| BMI(kg/m^2^）Tertile |  |  |  |
| Low | 1135（32.72%） | 0 | 0 |
| Medium | 1176（33.90%） | 4.37（3.06，5.68）<0.0001 | 1.74（0.82，2.66）0.0002 |
| High | 1158（33.38%） | 5.65（4.33，6.96）<0.0001 | 2.71（1.78，3.63）<0.0001 |
| Gender |  |  |  |
| Male | 1686（48.60%） | 0 | 0 |
| Female | 1783（51.40%） | -5.08（-6.15，-4.02）<0.0001 | -2.91（-3.66，-2.17）<0.001 |
| Age (years) |  |  |  |
| <60 | 2758（79.50%） | 0 | 0 |
| > =60 | 711（20.50%） | 13.36（12.10，14.62）<0.0001 | -2.39（-3.32，-1.46）<0.0001 |
| Race/Hispanic Origin |  |  |  |
| Mexican American | 656（18.91%） | 0 | 0 |
| Other Hispanic | 468（13.49%） | 0.58（-1.33，2.49）0.5514 | 0.11（-1.23，1.45）0.8717 |
| Non-Hispanic White | 1158（33.38%） | -0.73（-2.28，0.81）0.3516 | 0.34(-0.74，1.42）0.5359 |
| Non-Hispanic Black | 615（17.73%） | 2.64（0.87，4.42）0.0035 | 1.72（0.47，2.96）0.0068 |
| Other Races（a） | 572（16.49%） | -3.32（-5.12，-1.51）0.0003 | 2.73（1.47，4.00）<0.0001 |
| Education level -Adults 20+ |  |  |  |
| Less than 9th grade | 370（10.67%） | 0 | 0 |
| 9-11th grade (b) | 385（11.10%） | -2.69（-4.98，-0.39）0.0216 | 0.39（-1.22，2.01）0.6336 |
| High school graduate (c) | 741（21.36%） | -2.70（-4.71，-0.70）0.0083 | -0.04（-1.45，1.37）0.9550 |
| Some college or AA degree | 1043（30.07%） | -5.37（-7.27，-3.46）<0.0001 | -0.37（-1.71，0.98）0.5911 |
| College graduate or above | 930（26.81%） | -6..99（-8.93，-5.06）<0.0001 | 0.52（-0.84，1.89）0.4510 |
| Marital Status |  |  |  |
| Married | 1783（51.40%） | 0 | 0 |
| Widowed | 144（4.15%） | 10.76（8.04，13.49）<0.0001 | -3.94（-5.85，-2.02）<0.0001 |
| Other | 1542（44.45%） | -1.16（-2.26，-0.07）0.0376 | -1.00（-1.77，-0.23）0.0107 |
| Smoking |  |  |  |
| Yes | 1386（39.95%） | 0 | 0 |
| No | 2083（60.05%） | -3.98（-5.08，-2.89）<0.0001 | -0.13（-0.90，-0.64）0.7485 |

Note:Continuous variables are expressed as mean ± SD; categorical variables are expressed as n (%). The first group serves as the reference for each univariate analysis group (β = 0); (a) includes multiracial; (b) includes 12th grade, no diploma; (c) GED or equivalent. Abbreviation：SBP: Systolic Blood Pressure; DBP: Diastolic Blood Pressure.β = Beta value, CI = Confidence Interval
