# Supplementary material for: Positive association between serum lactate dehydrogenase levels and blood pressure: evidence from NHANES 2015–2016
Source: Front Cardiovasc Med. 2025 Feb 28;12:1554702. doi: 10.3389/fcvm.2025.1554702 (PMC11906999; doi:10.3389/fcvm.2025.1554702)
Supplement: Supplementary file 5 [file Table3.docx]

**Table S3: Continuation of stratified analysis between serum lactate dehydrogenase and baseline SBP, serum lactate dehydrogenase and baseline DBP**

| **Sub-group**  **X= Lactate Dehydrogenase (IU/L)** **Tertile** | **SBP(mmHg)**  **β(95%CI) *p*-value** | **DBP(mmHg)**  **β(95%CI) *p*-value** |
| --- | --- | --- |
| Albumin (g/L) Tertile |  |  |
| Low |  |  |
| Low | 0 | 0 |
| Medium | 3.18（0.52,5.84） 0.0195 | 2.49（0.68,4.31）0.0073 |
| High | 10.17（7.55,12.80）<0.0001 | 3.76（1.97,5.55）<0.0001 |
| Medium |  |  |
| Low | 0 | 0 |
| Medium | 4.89（2.65,7.13）<0.0001 | 2.28（0.70,3.86）0.0049 |
| High | 7.74（5.55,9.92）<0.0001 | 2.65（1.11,4.20）0.0008 |
| High |  |  |
| Low | 0 | 0 |
| Medium | 3.83（1.84,5.83）0.0002 | 2.69（1.23,4.15）0.0003 |
| High | 7.77（5.77,9.77）<0.0001 | 3.96（2.49,5.43）<0.0001 |
| Blood Urea Nitrogen (mmol/L) Tertile |  |  |
| Low |  |  |
| Low | 0 | 0 |
| Medium | 2.24（0.17,4.32）0.0344 | 2.17（0.57,3.76）0.0078 |
| High | 7.14（5.00,9.29）<0.0001 | 4.19（2.54,5.84）<0.0001 |
| Medium |  |  |
| Low | 0 | 0 |
| Medium | 3.85（1.38,6.32）0.0023 | 2.97（1.25,4.70）0.0008 |
| High | 7.92（5.50,10.34）<0.0001 | 3.34（1.64,5.03）0.0001 |
| High |  |  |
| Low | 0 | 0 |
| Medium | 5.17（2.92,7.42）<0.0001 | 2.32（0.80,3.84）0.0029 |
| High | 9.08（6.90,11.27）<0.0001 | 2.83（1.35,4.31）0.0002 |
| Bicarbonate (mmol/L) Tertile |  |  |
| Low |  |  |
| Low | 0 | 0 |
| Medium | 4.26（2.12,6.41）<0.0001 | 4.07（2.49,5.65）<0.0001 |
| High | 8.00（5.82,10.17）<0.0001 | 4.64（3.04,6.24）<0.0001 |
| Medium |  |  |
| Low | 0 | 0 |
| Medium | 6.49（3.66,9.32）<0.0001 | 2.23（0.08,4.37）<0.0001 |
| High | 9.80（7.00,12.59）<0.0001 | 3.42（1.29,5.54）<0.0001 |
| High |  |  |
| Low | 0 | 0 |
| Medium | 2.66（0.67,4.65）0.0089 | 1.50（0.15,2.85）<0.0001 |
| High | 7.92（5.97,9.86）<0.0001 | 2.65（1.33,3.96）<0.0001 |
| Total calcium(mmol/L) Tertile |  |  |
| Low |  |  |
| Low | 0 | 0 |
| Medium | 3.99（1.56,6.42）0.0014 | 3.83（2.17,5.49）<0.0001 |
| High | 10.99（8.57,13.40）<0.0001 | 5.28（3.63,6.92）<0.0001 |
| Medium |  |  |
| Low | 0 | 0 |
| Medium | 4.63（2.49,6.78）<0.0001 | 1.82（0.27,3.36）0.0215 |
| High | 7.45（5.35,9.54）<0.0001 | 2.53（1.02,4.04）<0.0001 |
| High |  |  |
| Low | 0 | 0 |
| Medium | 3.05（0.84,5.27）0.0070 | 1.96（0.36,3.56）0.0167 |
| High | 6.91（4.69,9.13）<0.0001 | 2.74（1.14,4.35）0.0008 |
| Chloride(mmol/L)Tertile |  |  |
| Low |  |  |
| Low | 0 | 0 |
| Medium | 1.37（-1.13,3.86）0.2829 | 0.90(-0.82,2.61）0.3059 |
| High | 6.89（4.45,9.34）<0.0001 | 3.11（1.43,4.79）0.0003 |
| Medium |  |  |
| Low | 0 | 0 |
| Medium | 5.10（2.85,7.35）<0.0001 | 2.20（0.53,3.86）0.0098 |
| High | 8.56（6.30,10.81）<0.0001 | 2.98（1.31,4.65）0.0005 |
| High |  |  |
| Low | 0 | 0 |
| Medium | 4.83（2.76,6.90）<0.0001 | 3.94（2.48,5.40）<0.0001 |
| High | 9.04（6.99,11.09）<0.0001 | 3.81（2.37,5.26）<0.0001 |
| Triglycerides  ,refrigserum (mmol/L) Tertile |  |  |
| Low |  |  |
| Low | 0 | 0 |
| Medium | 4.08（1.90,6.26）0.0003 | 1.32（-0.15,2.78）0.0783 |
| High | 11.39（9.18,13.59）<0.0001 | 3.20（1.72,4.68）<0.0001 |
| Medium |  |  |
| Low | 0 | 0 |
| Medium | 3.25（0.99,5.51）0.0049 | 3.17（1.59,4.75）<0.0001 |
| High | 6.74（4.51,8.98）<0.0001 | 2.73（1.17,4.29）0.0006 |
| High |  |  |
| Low | 0 | 0 |
| Medium | 2.96（0.67,5.25）<0.0001 | 2.28（0.55,4.00）0.0097 |
| High | 5.58（3.34,7.82）<0.0001 | 3.44（1.75,5.12）<0.0001 |
| Uric acid(umol/L) Tertile |  |  |
| Low |  |  |
| Low | 0 | 0 |
| Medium | 4.26（2.18,6.33）<0.0001 | 2.67（1.23,4.12）0.0003 |
| High | 8.62（6.43,10.81）<0.0001 | 2.92（1.39,4.44）0.0002 |
| Medium |  |  |
| Low | 0 | 0 |
| Medium | 3.01（0.73,5.28）0.0097 | 2.35（0.85,3.86）0.0023 |
| High | 8.82（6.55,11.08）<0.0001 | 4.15（2.65,5.65）<0.0001 |
| High |  |  |
| Low | 0 | 0 |
| Medium | 3.63（1.24,3.01）0.0030 | 1.96（0.13,3.79）0.0365 |
| High | 5.74（3.48,7.99）<0.0001 | 1.97（0.24,3.70）0.0262 |
| Creatinine（umol/L) Tertile |  |  |
| Low |  |  |
| Low | 0 | 0 |
| Medium | 3.14（0.97,5.31）0.0047 | 1.62（0.14,3.09）0.0321 |
| High | 7.38（5.14,9.62）<0.0001 | 1.96（0.44,3.49）0.0117 |
| Medium |  |  |
| Low | 0 | 0 |
| Medium | 2.59（0.49,4.68）0.0159 | 2.28（0.71,3.84）0.0046 |
| High | 9.23（7.12,11.35）<0.0001 | 4.14（2.55,5.72）<0.0001 |
| High |  |  |
| Low | 0 | 0 |
| Medium | 5.61（3.13,8.08）<0.0001 | 3.53（1.77,5.29）<0.0001 |
| High | 7.28（4.93,9.63）<0.0001 | 3.65（1.98,5.32）<0.0001 |
| Aspartate aminotransferase AST(U/L) Tertile |  |  |
| Low |  |  |
| Low | 0 | 0 |
| Medium | 4.84（2.77，6.92）<0.0001 | 1.75（0.32，3.18）0.0163 |
| High | 9.08（6.36，11.80）<0.0001 | 1.89（0.02，3.77）0.0476 |
| Medium |  |  |
| Low | 0 | 0 |
| Medium | 3.13（0.93，5.33）0.0054 | 2.44（0.87，4.01）0.0024 |
| High | 6.90（4.66，9.15）<0.0001 | 2.51（0.91，4.11）0.0022 |
| High |  |  |
| Low | 0 | 0 |
| Medium | 1.31（-1.47，4.10）0.3551 | 2.43（0.42，4.43）0.0179 |
| High | 5.59（3.05，8.14）<0.0001 | 3.17（1.34，5.00）0.0007 |
| Alanine aminotransferase ALT(U/L) Tertile |  |  |
| Low |  |  |
| Low | 0 | 0 |
| Medium | 5.59（3.25，7.92）<0.0001 | 1.88（0.34，3.42）0.0169 |
| High | 10.20（7.45，12.94）<0.0001 | 1.68（-0.13，3.49）0.0695 |
| Medium |  |  |
| Low | 0 | 0 |
| Medium | 3.21（1.04，5.37）0.0037 | 1.70（0.19，3.21）0.0274 |
| High | 7.83（5.68，9.97）<0.0001 | 2.80（1.30，4.30）0.0003 |
| High |  |  |
| Low | 0 | 0 |
| Medium | 1.19（-1.18，3.56）0.3253 | 2.72（0.95，3.21）0.0274 |
| High | 5.59（3.05，8.14）<0.0001 | 2.74（1.08，4.41）0.0013 |
| Body Mass Index(BMI) (kg/m^2^）Tertile |  |  |
| Low |  |  |
| Low | 0 | 0 |
| Medium | 3.22（1.11,5.32）0.0028 | 1.53（0.10,2.97）0.0359 |
| High | 8.78（6.54,11.03）<0.0001 | 3.40（1.87,4.93）<0.0001 |
| Medium |  |  |
| Low | 0 | 0 |
| Medium | 4.45（2.05,6.85）0.0003 | 2.46（0.80,4.11）0.0037 |
| High | 7.76（5.37,10.15）<0.0001 | 2.54（0.90,4.19）0.0025 |
| High |  |  |
| Low | 0 | 0 |
| Medium | 3.09（0.84,5.34）0.0072 | 3.31（1.57,5.04）0.0002 |
| High | 6.79（4.66,8.92）<0.0001 | 3.75（2.21,5.39）<0.0001 |
| Gender |  |  |
| Male |  |  |
| Low | 0 | 0 |
| Medium | 2.64（0.76，4.52）0.0059 | 2.49（1.07,3.92）0.0006 |
| High | 5.61（3.79，7.44）<0.0001 | 3.77（2.38,5.15）<0.0001 |
| Female |  |  |
| Low | 0 | 0 |
| Medium | 4.63（2.85，6.41）<0.0001 | 2.37（1.18,3.56）<0.0001 |
| High | 10.29（8.49，12.09）<0.0001 | 2.75（1.55,3.95）<0.0001 |
| Age (years) |  |  |
| <60 |  |  |
| Low | 0 | 0 |
| Medium | 3.16（1.88，4.44）<0.0001 | 2.67（1.68,3.66）<0.0001 |
| High | 7.53（6.23，8.83）<0.0001 | 4.44（3.43,5.44）<0.0001 |
| > =60 |  |  |
| Low | 0 | 0 |
| Medium | 3.24（-0.45，3.94）0.0860 | 2.39（-0.05,4.84）0.0549 |
| High | 3.89（0.43，7.34）0.0277 | 1.72（-0.56,4.00）0.1391 |
| Race/Hispanic Origin |  |  |
| Mexican American |  |  |
| Low | 0 | 0 |
| Medium | 3.08（0.18，5.98）0.0376 | 1.77（-0.16,3.70）0.0432 |
| High | 5.66（2.70，8.62）0.0002 | 3.56（1.60,5.53）0.0004 |
| Other Hispanic |  |  |
| Low | 0 | 0 |
| Medium | 4.39（0.76，8.02）0.0181 | 3.82（1.27,6.37）0.0035 |
| High | 10.13（6.59，13.68）<0.0001 | 3.76（1.27,6.26）0.0032 |
| Non-Hispanic White |  |  |
| Low | 0 | 0 |
| Medium | 4.14（1.97，6.30）0.0002 | 2.67（1.10,4.24）0.0009 |
| High | 7.51（5.29，9.73）<0.0001 | 2.12（0.51,3.74）0.0099 |
| Non-Hispanic Black |  |  |
| Low | 0 | 0 |
| Medium | 4.71（1.07，8.35）0.0114 | 3.07（0.53,5.60）0.0181 |
| High | 10.58（7.22，13.93）<0.0001 | 5.17（2.84,7.51）<0.0001 |
| Other Races（a） |  |  |
| Low | 0 | 0 |
| Medium | 3.30（0.38，6.22）0.0272 | 1.81（-0.39,4.01）0.1073 |
| High | 8.19（5.32，11.06）<0.0001 | 3.79（1.63,5.96）0.0006 |
| Education level -Adults 20+ |  |  |
| Less than 9th grade | 0 | 0 |
| Low | 5.18（0.56,9.81）0.0287 | 0.77（-2.14,3.68）0.6054 |
| Medium | 8.00（3.50,12.50）0.0006 | 2.96（0.13,5.79）0.010 |
| High |  |  |
| 9-11th grade (b) |  |  |
| Low | 0 | 0 |
| Medium | 4.14（-0.08,8.35）0.0454 | 4.20（1.39,7.01）0.0036 |
| High | 8.01（392,12.10）0.0001 | 4.64（1.92,7.37）0.0009 |
| High school graduate (c) |  |  |
| Low | 0 | 0 |
| Medium | 3.20（0.15,6.25）0.0404 | 2.90（0.79,5.02）0.0074 |
| High | 7.83（4.84,10.81）<0.0001 | 2.95（0.88,5.02）0.0053 |
| Some college or AA degree |  |  |
| Low | 0 | 0 |
| Medium | 4.18（1.94,6.42）0.0003 | 3.30（1.58,5.02）0.0002 |
| High | 8.07（5.84,10.31）<0.0001 | 3.92（2.20,5.64）<0.0001 |
| College graduate or above |  |  |
| Low | 0 | 0 |
| Medium | 3.22（1.00,5.05）0.0046 | 1.47（-0.17,3.11）0.0800 |
| High | 8.62（6.37,10.86）<0.0001 | 3.25（1.59,4.91）0.0001 |
| Marital status |  |  |
| Married |  |  |
| Low | 0 | 0 |
| Medium | 3.25（1.45,5.05）0.0004 | 1.88（0.60,3.15）0.0039 |
| High | 7.61（5.83,9.40）<0.0001 | 2.82（1.55,4.09）0.0001 |
| Widowed |  |  |
| Low | 0 | 0 |
| Medium | 6.14（-1.31,13.59）0.1086 | 2.46（0.80,4.11）0.0037 |
| High | 6.06（-0.78,12.90）0.0846 | 2.54（0.90,4.19）0.0025 |
| Other |  |  |
| Low | 0 | 0 |
| Medium | 4.29（2.36,6.22）<0.0001 | 3.31（1.57,5.04）0.0002 |
| High | 8.86（6.37,10.86）<0.0001 | 3.75（2.11,5.39）<0.0001 |
| smoking |  |  |
| Yes |  |  |
| Low | 0 | 0 |
| Medium | 3.20（1.01,5.39）0.0043 | 3.18（1.62,4.74）<0.0001 |
| High | 7.47（5.31,9.64）<0.0001 | 2.78（1.24,4.32）0.0004 |
| No |  |  |
| Low | 0 | 0 |
| Medium | 4.21（2.61,5.81）<0.0001 | 2.07（0.93,3.21）0.0004 |
| High | 8.75（7.16,10.33）<0.0001 | 3.96（2.83,5.09）<0.0001 |

Note: (a) Including Multi-Racial; (b)Includes 12th grade with no diploma; (c) GED or equivalent. Weighted by: Full sample mobile examination center exam weight.Abbreviation:SBP: Systolic Blood Pressure; DBP: Diastolic Blood Pressure.β = Beta value, CI = Confidence Interval
