# Supplementary material for: Positive association between serum lactate dehydrogenase levels and blood pressure: evidence from NHANES 2015–2016
Source: Front Cardiovasc Med. 2025 Feb 28;12:1554702. doi: 10.3389/fcvm.2025.1554702 (PMC11906999; doi:10.3389/fcvm.2025.1554702)
Supplement: Supplementary file 6 [file Table4.docx]

**Table S4.Threshold and Saturation Effect Analysis**

| **Results** | **SBP(mmHg）**  **β (95%CI) *p*-value** | **DBP(mmHg）**  **β (95%CI) *p*-value** |
| --- | --- | --- |
| Model I |  |  |
| Linear Effect | 0.053（0.032，0.074）<0.0001 | 0.031（0.015，0.047）0.0002 |
| Model II |  |  |
| Breakpoint (K) | 115 | 123 |
| Effect 1 (<K) | 0.090（0.028，0.151）0.0043 | 0.079（0.042，0.115）<0.0001 |
| Effect 2 (>K) | 0.042（0.015，0.069）0.0021 | 0.007（-0.017，0.030）0.5739 |
| Difference in Effects Between 2 and 1 | -0.048（-0.123，0.027）0.2128 | -0.072（-0.121，-0.023）0.0043 |
| Predicted Value at the Breakpoint | 120.866（120.049，121.683） | 70.804（70.218，71.390） |
| Log-Likelihood Ratio Test | 0.211 | 0.004 |

Abbreviation:SBP: Systolic Blood Pressure; DBP: Diastolic Blood Pressure.β = Beta value, CI = Confidence Interval.Weighting basis: Complete sample with mobile examination center examination weights. Outcome variables: Baseline SBP; Baseline DBP. Exposure variable: Lactate Dehydrogenase (U/L).Adjusted for age, sex, race/Hispanic origin, marital status, Education level-Adults 20+, smoking status, albumin, total calcium, blood urea nitrogen, bicarbonate, chloride, triglycerides, uric acid, creatinine, aspartate aminotransferase (AST), alanine aminotransferase (ALT). When the P-value in Model I is less than 0.05, the model shows a linear effect. When the P-value in Model I is greater than 0.05, the model exhibits a segmented effect in Model II, with K being the lactate dehydrogenase level at the inflection point; β represents the slope of the curve, and segments with β P-value less than 0.05 are statistically significant. The K value is the turning point, which is the level of lactate dehydrogenase at which the relationship between lactate dehydrogenase and systolic and diastolic blood pressure changes.
